# Evaluation of Side Effects Associated with COVID-19 Vaccines in Saudi Arabia

**DOI:** 10.3390/vaccines9060674

**Published:** 2021-06-18

**Authors:** Abdulaziz Alhazmi, Edrous Alamer, Dalia Daws, Mashael Hakami, Majid Darraj, Siddig Abdelwahab, Amani Maghfuri, Abdullah Algaissi

**Affiliations:** 1 Department of Microbiology and Parasitology, College of Medicine, Jazan University, Jazan 45142, Saudi Arabia; abalhazmi@jazanu.edu.sa; 2 Jazan University Hospital, Jazan University, Jazan 45142, Saudi Arabia; ddous@jazanu.edu.sa (D.D.); mashael@jazanu.edu.sa (M.H.); 3 Medical Research Center, Jazan University, Jazan 45142, Saudi Arabia; ealamer@jazanu.edu.sa (E.A.); sadiqa@jazanu.edu.sa (S.A.); 4 Department of Medical Laboratories Technology, College of Applied Medical Sciences, Jazan University, Jazan 45142, Saudi Arabia; 5 Department of Medicine, College of Medicine, Jazan University, Jazan 45142, Saudi Arabia; mdarraj@jazanu.edu.sa; 6 College of Medicine, Jazan University, Jazan 45142, Saudi Arabia; Amanimgf@gmail.com

**Keywords:** COVID-19, SARS-CoV-2, vaccine, side effects

## Abstract

Background: Pfizer-BioNTech and Oxford-AstraZeneca are recently introduced vaccines to combat COVID-19 pandemic. During clinical trials, mild to moderate side effects have been associated with these vaccines. Thus, we aimed to evaluate short-term post-vaccination side effects. Methods: Cross-sectional, retrospective study using an online questionnaire was conducted among COVID-19 vaccines recipients in Saudi Arabia. General and demographic data were collected, and vaccine-associated side effects after receiving at least one dose of each vaccine were evaluated. Results: Our final sample consisted of 515 participants with a median age of 26 years. Most of the study participants were female (57%). Nearly 13% of the study subjects have reported previous infections with SARS-CoV-2. Oxford-AstraZeneca and Pfizer-BioNTech vaccines have been received by 75% and 25% of the study participants, respectively. Side effects associated with COVID-19 vaccines have been reported by 60% of the study subjects, and most of them reported fatigue (90%), pain at the site of the injections (85%). Conclusion: Side effects that are reported post Oxford-AstraZeneca and Pfizer-BioNTech vaccines among our study participants are not different from those that were reported in the clinical trials, indicating safe profiles for both vaccines. Further studies are needed to evaluate the effectiveness of the current vaccines in protection against SARS-CoV-2 reinfections.

## 1. Introduction

Severe acute respiratory syndrome coronavirus 2 (SARS-CoV-2), is a newly identified member of the human coronaviruses family that was discovered during the outbreak of the highly transmissible respiratory disease in Wuhan, China in 2019. SARS-CoV-2 causes the Coronavirus disease 19 (COVID-19) and is now continuing to spread worldwide causing a global pandemic [1]. Saudi Arabia is one of the countries that was affected by this pandemic. More importantly, Saudi health officials have taken early unprecedented preventive measures and precautionary strategies, such as banning international flights, closing mosques, schools, and universities, and complete lockdown of the country, to reduce the burden of the disease [2]. Yet, as there is no approved antiviral treatment for COVID-19, several trials for vaccine development were immediately initiated with the hope to control this pandemic [3,4]. By the beginning of 2021, some international health authorities announced various vaccine candidates for emergency use authorization (EUA) [5,6,7]. Pfizer-BioNTech mRNA vaccine (BNT162b2) and Oxford-AstraZeneca vaccine (ChAdOx1 nCoV-19) were the first vaccines that were approved and introduced to Saudi Arabia. The initial authorization for these vaccines was attained for some high-risk and vulnerable groups, such as healthcare workers and old people with chronic diseases, then they became widely available for the whole population, excluding children and pregnant women [8]. Pfizer-BioNTech vaccine (BNT162b2) is based on the mRNA technology to express the SARS-CoV-2 spike (S) gene and has shown a high efficacy rate against SARS-CoV-2 infection. Specifically, phase III trials showed that BNT162b2 has about 95% efficacy against laboratory-confirmed SARS-CoV-2 symptomatic infection, at least seven days after the second dose in the individual of 16 years and older without current or previous history of COVID-19. mRNA vaccines are a new type of vaccine that has been recently utilized. BNT162b2 mRNA vaccine has been developed to stimulate immune response against SARS-CoV-2 using mRNA coding SARS-CoV-2 spike protein [8,9]. This vaccine was approved by the U.S. food and drug administration (FDA) on the 11th of December 2020 for EUA in individuals older than 16 years of age. Consequently, the Saudi food and drug administration (SFDA) approved BNT162b2 to be used in Saudi Arabia. On the other hand, two doses of Oxford-AstraZeneca adenovirus-vectored vaccine (ChAdOx1 nCoV-19) showed an overall 63% efficacy against symptomatic SARS-CoV-2 infection. This vaccine was authorized to be used in the age group of 18 years and older. Unlike BNT162b2, ChAdOx1 nCoV-19 uses replication-deficient chimpanzees adenovirus as a viral vector to express the SARS-COV-2 spike protein [8,10]. Clinical trials have shown that both vaccines were associated with various mild to moderate side effects, such as pain, redness or swelling at the site of injection, tiredness, headaches, chills, muscle, and joint aches, and fever [9,10].

In our study, we evaluated the short-term side effects after receiving either Pfizer-BioNTech mRNA (BNT162b2) or Oxford-AstraZeneca (ChAdOx1 nCoV-19) vaccines in a sample of 18 years and older citizens and residents of Saudi Arabia.

## 2. Materials and Methods

This is a retrospective, cross-sectional, online survey that was conducted over a period of 3 weeks, between 7 April and 28 April 2021, in Saudi Arabia. The vaccination of the general population against COVID-19 in Saudi Arabia was initiated in early 2021 for both Pfizer-BioNTech and Oxford-AstraZeneca vaccines. A dual language (Arabic and English) online questionnaire was designed using Google Forms which was delivered to participants via social media. Additionally, an electronic email was also established and used to facilitate communication between study investigators and participants. Investigators kept careful and frequent verification for the participants’ responses. The study questionnaire was divided into two main sections. The first section was designed to collect general information about the participants such as gender, age, chronic diseases, and infection status with SARS-CoV-2. The second section focused on the COVID-19 vaccine-related data such as type and date of COVID-19 vaccine, the first or second doses, side effects that are commonly associated with the COVID-19 vaccine, timing, and duration of the side effects. In our side effects subsection, we have included the most common side effects which were reported in other studies [8,9,10], including pain at the sites of injections, fatigue, headache, fever and chills, nausea, and vomiting, in addition, we also provided a section for reporting other unlisted side effects which may be experienced by our study participants. Doctors’ visits after vaccination and admission to the hospital post-vaccination were also included in our study questionnaire. Furthermore, participants were also questioned to report any medications taken post-vaccination and this parameter was evaluated during our analysis to exclude this major confounding factor. The ethical approval committee at Jazan University approved this study (reference number; REC42/1/087, date 22 March 2021) and consents have been taken from all participants. This study included participants who received either single or double doses of Pfizer-BioNTech or Oxford-AstraZeneca vaccines in Saudi Arabia. We have excluded all participants who refused to participate or those who were not vaccinated against COVID-19 or who had received a vaccine other than Pfizer-BioNTech and Oxford-AstraZeneca vaccines. The sample size was calculated using raosoft.com. The ministry of health in Saudi Arabia initially aimed to vaccinate 70% of the population (about 28 million) [11]. Consequently, a sample size of 385 was determined to be sufficient to give a 95% confidence interval with a 5% margin of error, for a population of 30 million. However, the sample size was increased by about 30% of participants in order to increase the statistical power of our study results as well as to reduce sampling bias in our method as we used an online questionnaire on social media. Descriptive statistics were reported for the collected data. *t*-test and chi-square test were done for statistical analysis. The data were statistically analyzed using SPSS v.23 (IBM Corp., Armonk, NY, USA) with a significance level of *p* ≤ 0.05. Multivariate logistic regression models were also applied to all symptoms that might be related to the side effects from which we obtained adjusted odds ratio (aOR), 95% confidence intervals (CI).

## 3. Results

### 3.1. Study Participants’ Demographics and General Characteristics

Five hundred thirty-three individuals have participated in this study, and 18 of them did not meet our inclusion criteria. Our final sample consists of 515 participants with a median age of 26 years (SD:9) ranging from 18 to 70 years, of whom 57% were female (*n* = 294). Thirteen percent (66) of the study participants have been previously diagnosed with COVID-19. About 86% of the participants denied having a history of any chronic diseases. Most of the participants received Oxford-AstraZeneca (75%), followed by Pfizer-BioNTech (25%). Only 39 (8%) of the participants have received two doses from the same vaccine [8]. Sixty percent (307) of the study subjects in our study have reported side effects due to the COVID-19 vaccine. These data are also summarized in Table 1. We also categorized our study participants into two main groups, i.e., participants who experienced side effects and participants who have not experienced similar side effects. These two groups were compared against each other using several parameters including age, sex, history of chronic diseases or any previous SARS-CoV-2 infection, type of vaccines, and the number of doses received yet (Table 2). The median age in our study participants did not differ significantly between the two groups (27 years vs. 26 years). Moreover, the participants who received two doses experienced significantly more side effects (10%) compared to the participants who did not report side effects (4%). Further, the side effects that were reported by the individuals who received Oxford-AstraZeneca vaccine were significantly more compared to the individuals who received Pfizer-BioNTech (83% and 17%, respectively).

### 3.2. Participants Who Reported Side Effects

Three hundred seven of our study participants (60%) experienced side effects due to the COVID-19 vaccines (Table 3). Nearly 84% reported side effects immediately on the day of receiving the vaccine, while 15% and 1% of the participants started to notice such side effects on the second- and third-day post-vaccination, respectively. The duration of the side effects lasted from one to three days for 75% of the participants, and from three to five days for 21% of them, with only 11% reported extended duration of the side effects (more than 5 days). In particular, fatigue and pain, and redness at the site of injection were the most commonly reported side effects among our study participants (90% and 85%, respectively) (Table 3). Sixty-six percent of the participants reported fever and 36% of them reported having chills. Headache was common among the individuals of our study (62%). However, nausea, vomiting, joint, and bone pain were less commonly reported by our study participants, (28% and 2%, respectively). Then, we used univariate and multivariate analyses to identify the most common side effects for each vaccine, i.e., Oxford-AstraZeneca and Pfizer-BioNTech (Table 4). We found that fatigue and fever (92% and 71%) were more significantly associated with the Oxford-AstraZeneca vaccine compared to Pfizer-BioNTech vaccine which showed 77% and 44%, respectively. However, no significant difference was found for other side effects.

## 4. Discussion

Since the beginning of the COVID-19 pandemic in January 2020, most countries have taken precautionary measures to control SARS-CoV-2 transmission with the hope of rapid production of safe and effective vaccines [2]. In response, different vaccine candidates have been simultaneously developed and only a few of them were authorized for EUA [5]. Saudi Arabia is one of the countries that have started an early vaccination campaign as a continuum for its early unprecedented efforts and actions to combat SARS-CoV-2 spread [2,8]. Despite the availability of the vaccine for the population in Saudi Arabia, there is a variation in people’s acceptance to take the vaccine and this is probably due to the fact that these vaccines were developed in a short time compared to the previously approved vaccines which usually take years before approval. Another reason for this variation could be related to the usage of a newly emerging technique for some of the COVID-19 vaccines, mRNA vaccines [12,13,14,15,16]. These two major factors may raise the concern among some individuals about potential severe post-vaccination side effects, although several reports describing the expected side effects have been issued recently [8]. Thus, in this study, we aimed to evaluate the short-term side effects associated with the COVID-19 vaccines which are currently used in Saudi Arabia. We collected data from individuals who received COVID-19 vaccines in Saudi Arabia, either Oxford-AstraZeneca or Pfizer-BioNTech vaccines. Both vaccines have reported 60% to 80% of the side effects associated with the vaccine and this variation due to the age of the individual, type, and the dose [9,10]. In our study, 60% of the participants reported some side effects. The most common side effects were fatigue (90%), pain at the site of injections (85%), followed by fever (66%), and headache (62%). Most of the participants reported having side effects on the first day upon receiving the vaccines (85%) with a one-day duration of the side effects (75%). These findings are highly consistent with the reported results in phase III clinical trials and fact sheets of the vaccines, and it’s mostly reported for those who received the second dose [9,10]. Similar data were also reported in a recent study conducted by Menni and his group [17]. This study found that tenderness and local pain around the injection site are the most commonly reported side effects, and it occurred on the same day after the injection and lasted for about one day [17]. Furthermore, in a study conducted on participants receiving Pfizer-BioNTech in Saudi Arabia, it was found that 70% to 80% of the study participants have reported pain at the site of injection [18]. Unlike other studies, most of the participants in our study reported having tiredness and headache, and this is mainly explained by the younger age of our participants (median is 26 years) compared to others’ findings [17,18]. As it’s also evidenced in several studies that younger individuals reported a higher frequency of side effects compared to older individuals [9,19]. For the same reason, i.e., younger age of the participants, neither age nor gender was significantly associated with side effects. For example, in Menni et al. study the mean age of the participants was 50 years, and the majority of them were older than 55 years, where they reported less frequency of tiredness, from 8% to 21% of the participants, and women experienced more side effects than men [17]. It’s known that individuals who were vaccinated with the Oxford-AstraZeneca vaccine are more likely to have systemic side effects, such as fatigue and fever, compared to those who received Pfizer-BioNTech vaccine [20]. In our study, we found that the participants who received the Oxford-AstraZeneca vaccine reported a significantly higher frequency of fatigue and headache than those who received the Pfizer-BioNTech vaccine (Table 4). It’s noteworthy that our study was conducted at the time when the Saudi ministry of health has decided to postpone the second dose [21], a decision that reflected on our study sample in which we included only 8% who received the second dose and most of them (70%) were in Pfizer-BioNTech vaccine group due to its shorter interval period between two doses. It would be interesting to include a larger sample of participants with the second dose to evaluate their experience of severe side effects compared to the first dose. However, with the current sample, we found a significant difference between the participants who experienced side effects by the second injection (10% vs. 4%, *p* 0.012). It has been reported, either from the trial results or the real-world data, that the side effects associated with the COVID-19 vaccine are mild to moderate [9,10,17,18]. In the current report, we found that only 3% of the participants needed to see a doctor due to the side effects of the vaccines, and 1% admitted to the hospital, a finding that can be reassuring for the concerned public about the safety of the available vaccines.

Despite it being one of the few studies in Saudi Arabia that has discussed the side effects related to COVID-19 vaccines, our study has many limitations. The data have been collected by a self-administered online questionnaire and this could result in a reporting bias. Because COVID-19 pandemic and the recommendation to continue the social distancing and the preventive measures in Saudi Arabia, we preferred to conduct this study as a web-based study to ensure the safety of all study participants. Moreover, community-based surveys would be difficult to be done during this pandemic. Therefore, data collection was online as a self-reported survey, and the distribution of this survey depending on the authors’ networks. As such, most of the participants were young. It would be insightful if we included more participants who benefited from the Pfizer-BioNTech vaccine, but the distribution process at the time of the study for the Pfizer-BioNTech vaccine was limited. Furthermore, it was not possible to assess the long-term side effects of the COVID-19 vaccine due to the inaccessibility of the online questionnaire to the participants. However, it would be helpful to assess the thromboembolic profiles and symptoms for the participants to evaluate its association with the current vaccines.

## 5. Conclusions

In this study, we assessed the short-term side effects associated with COVID-19 vaccines approved for use in Saudi Arabia, Oxford-AstraZeneca, and Pfizer-BioNTech vaccines. We found that most of the participants reported fatigue, pain at the site of the injection, fever, and headache, and they are more common in those after the second dose of the vaccines. Moreover, only a few patients needed to see a doctor or to be admitted to the hospital due to vaccines’ side effects. Fatigue and fever were significantly associated with Oxford-AstraZeneca, compared to the Pfizer-BioNTech vaccine. A follow-up study on a larger population is warranted to evaluate the effectiveness of the vaccines on the control and prevention of SARS-CoV-2 infection as well as the long-term side effects.

## Figures and Tables

**Table 1:** General characteristics of the participants in our study.

Characteristic	Participants, *n* = 515
Age, years (Median; SD)	26; 9
Male, *n* (%)	221 (43%)
History of COVID-19, *n* (%)	66 (13%)
History of chronic diseases, *n* (%)	71 (14%)
Pfizer-BioNTech Oxford-AstraZeneca	130 (25%) 385 (75%)
Received two doses, *n* (%)	39 (8%)
Experienced side effects, *n* (%)	307 (60%)

SD: Standard deviation.

**Table 2:** Univariate analysis of the participants who presented with side effects compared to those without side effects due to COVID-19 vaccination.

Characteristic	Participants with Side Effects, *n* = 307 (60%)	Participants with no Side Effects, *n* = 208 (40%)	*p*-Value ^#^
Age, years (Median; SD)	27; 8	26; 10	0.209
Male, *n* (%)	125 (41%)	95 (46%)	0.176
History of chronic diseases, *n* (%)	40 (13%)	31 (15%)	0.519
History of COVID-19, *n* (%)	44 (14%)	20 (9%)	0.810
Received two doses, *n* (%)	30(10%)	9(4%)	0.012 *
Pfizer-BioNTech Oxford-AstraZeneca	52 (17%) 255 (83%)	78 (38%) 130(62%)	<0.0001 *

SD: Standard deviation. ^#^ The alpha criterion for *p*-value was set to 0.05. * Significant in univariate analysis.

**Table 3:** General characteristics of the participants presented with side effects due to COVID-19 vaccination.

Characteristic	Participants with Side Effects, *n* = 307
Time of the side effects started to appear	
First day	257 (84%)
Second day	47 (15%)
Third day or later	3 (1%)
Duration of the side effects (days)	
From 1 to 3	231(75%)
From 3 to 5	65 (21%)
More than 5	11 (4%)
Taking medication to mitigate side effects	262(85%)
Visiting a physician due to side effects	21 (7%)
Hospitalization due to side effect	3 (1%)
Fatigue	274 (90%)
Pain or redness at the site of injection	261 (85%)
Fever	204 (66%)
Chills	111 (36%)
Headache	191 (62%)
Nausea or vomiting	87 (28%)
Joint or bone pain	5 (2%)

SD: Standard deviation.

**Table 4:** Univariate and multivariate analyses testing different side effects significantly associated with Oxford-AstraZeneca and Pfizer-BioNTech COVID19 vaccines.

Side effect	Univariate Analysis ^&^			Multivariate Analysis ^&&^	
	Oxford-AstraZeneca, *n* = 255 (83%)	Pfizer-BioNTech, *n* = 52 (17%)	*p*-Value ^#^	aOR	95% CI
Fatigue	236 (92%)	39 (75%)	0.001 *	0.289 **	0.124–0.671
Pain or redness at the site of injection	217 (85%)	44 (85%)	1	-	-
Fever	181 (71%)	23 (44%)	<0.0001 *	0.317 **	0.158–0.639
Chills	96 (38%)	15 (29%)	0.269	-	-
Headache	159 (62%)	32 (62%)	1	-	-
Nausea or vomiting	71 (28%)	16 (31%)	0.736	-	-

SD: Standard deviation. aOR: adjusted odds ratio. CI: confidence interval. ^#^ The alpha criterion for p-value was set to 0.05. * Significant in univariate analysis. ** Significant in multivariate analysis. ^&^ Chi-squared test and *t*-test were used for univariate analysis. ^&&^ Multiple logistic regression was used for multivariate analysis using the symptoms significantly associated with the Oxford-AstraZeneca vaccine.

## Data Availability

The data presented in this study are available on request from the corresponding author.
